# Bis(4-methylpiperidine-1-carbodithioato)-lead(II) and Bis(4-benzylpiperidine-1-carbodithioato)-lead(II) as Precursors for Lead Sulphide Nano Photocatalysts for the Degradation of Rhodamine B

**DOI:** 10.3390/molecules26237251

**Published:** 2021-11-29

**Authors:** Thandi B. Mbuyazi, Peter A. Ajibade

**Affiliations:** School of Chemistry and Physics, University of KwaZulu-Natal, Private Bag X01, Scottsville, Pietermaritzburg 3209, South Africa; 216006335@stu.ukzn.ac.za

**Keywords:** lead(II) dithiocarbamate, crystal structure, lead sulfide, nanophotocatalyst, photodegradation, rhodamine B

## Abstract

Bis(4-methylpiperidine-1-carbodithioato)-lead(II) and bis(4-benzylpiperidine-1-carbodithioato)-lead(II) were prepared and their molecular structures elucidated using single crystal X-ray crystallography and spectroscopic techniques. The compounds were used as precursors for the preparation of lead sulphide nano photocatalysts for the degradation of rhodamine B. The single crystal structures of the lead(II) dithiocarbamate complexes show mononuclear lead(II) compounds in which each lead(II) ion coordinates two dithiocarbamato anions in a distorted tetrahedral geometry. The compounds were thermolyzed at 180 ℃ in hexadecylamine (HDA), octadecylamine (ODA), and trioctylphosphine oxide (TOPO) to prepare HDA, ODA, and TOPO capped lead sulphide (PbS) nanoparticles. Powder X-ray diffraction (pXRD) patterns of the lead sulphide nanoparticles were indexed to the rock cubic salt crystalline phase of lead sulphide. The lead sulphide nanoparticles were used as photocatalysts for the degradation of rhodamine B with ODA-PbS1 achieving photodegradation efficiency of 45.28% after 360 min. The photostability and reusability studies of the as-prepared PbS nanoparticles were studied in four consecutive cycles, showing that the percentage degradation efficiency decreased slightly by about 0.51–1.93%. The results show that the as-prepared PbS nanoparticles are relatively photostable with a slight loss of photodegradation activities as the reusability cycles progress.

## 1. Introduction

Lead sulphide (PbS) nanomaterials are IV-VI semiconductor compounds with a large excitonic Bohr radius of 18 nm and narrow band gap of 0.41 eV at ambient temperature (300 K) [1]. In addition, they have a dielectric constant of 169 at 300 k as well as high carrier mobility [2]. PbS nanoparticles are used in a variety of fields, such as opto-electronic devices, solar concentrators, diodes, photodetectors, photovoltaic cells, sensors, thermoelectrics, and catalysts. PbS nanoparticles with different morphologies, such as nanorods [3], nanosheets [4], flower-like [5], nanocubes [6], star-shaped [7], and dendrites [8], have been synthesized using various methods. These methods include solvothermal [9], electrochemical deposition, spray pyrolysis, sonochemical [10], chemical bath deposition [11], gamma-ray irradiation, and single-source molecular precursor approaches [12,13,14,15].

Transition metal dithiocarbamates are widely used as single source precursors (SSPs) for the preparation of different metal sulphide nanomaterials due to their ease of synthesis and the dependence of their volatility and decomposition properties on the amine substituents [12]. Moreover, transition metal dithiocarbamate complexes have been found to decompose efficiently, resulting in metal sulphide nanoparticles with few or no impurities. It has been shown that the particle size and structural morphology of PbS nanoparticles depend on the preparation methods and the type of capping agents used for surface passivation, and all these determine the potential applications of the nanoparticles [13]. Studies have shown that the electrostatic and stabilizing effects of capping agents affect the growth of nanoparticles [14,15], and capping agents play significant role in the monodispersity of the as-prepared nanoparticle [16,17,18,19].

Herein, the preparation, single crystal structures, and spectroscopic studies of bis(4-methylpiperidine-1-carbodithioato)-lead(II) and bis(4-benzylpiperidine-1-carbodithioato)-lead(II) dithiocarbamate complexes are presented. The compounds were thermolyzed in hexadecylamine (HDA), octadecylamine (ODA), and trioctylphosphine oxide (TOPO) to prepare HDA, ODA, and TOPO capped lead sulphide (PbS) nanoparticles. The effect of the different capping agents and the precursors on the structural, morphological properties of the as-prepared lead sulphide nanoparticles were evaluated. The as-prepared lead sulphide nanoparticles were used as nano photocatalysts for the photocatalytic degradation of rhodamine B dye under visible light. 

## 2. Results and Discussion

### 2.1. Sybtheses

The ligands sodium salt of 4-methylpiperidine dithiocarbamate and 4-benzylpiperidine dithiocarbamate were prepared by the reaction of aqueous solution of sodium hydroxide or 4-methylpiperidine and 4-benzylpiperidine with cold carbon disulfide at 4 °C. The ligands were soluble in water, methanol, and ethanol with high yield for sodium salt of 4-methylpiperidine dithiocarbamate but low yield for the sodium salt of 4-benzylpiperidine dithiocarbamate. 

**Scheme 1:**
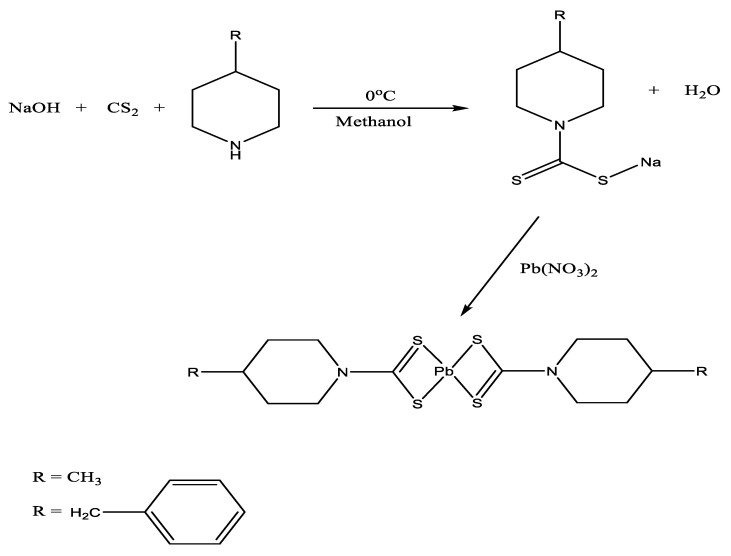
Preparation of the dithiocarbamate ligands and the lead(II) dithiocarbamate complexes.

The complexes were prepared (Scheme 1) by reacting the respective ligands with lead nitrate in aqueous solution to obtain air stable products with 92% yield that are soluble in chloroform and partially soluble in dimethyl sulfoxide and dichloromethane, but insoluble in all other solvents.

### 2.2. Molecular Structure of Bis(4-methylpiperidine-1-carbodithioato)-lead(II) and Bis(4-benzylpiperidine-1-carbodithioato)-lead(II)

**Figure 1:**
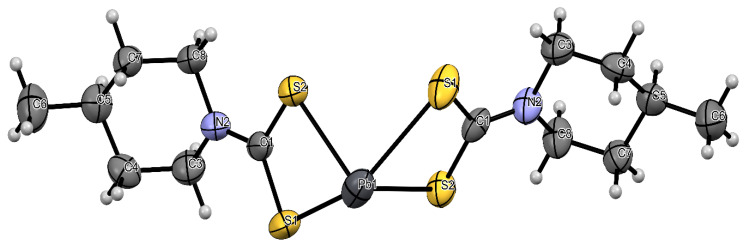
Molecular structure of bis(4-methylpiperidine-1-carbodithioato)-lead(II).

**Figure 2:**
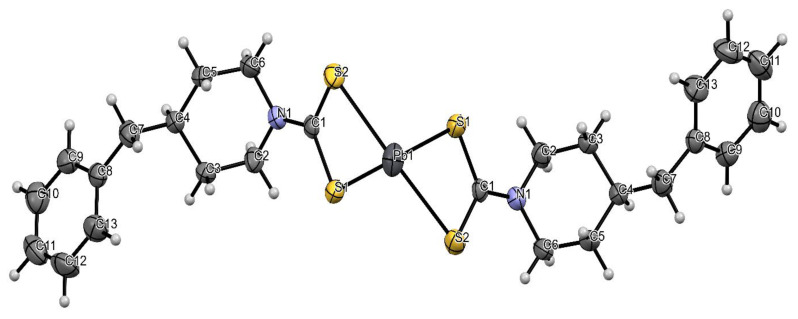
Molecular structure of bis(4-benzylpiperidine-1-carbodithioato)-lead(II).

**Figure 3:**
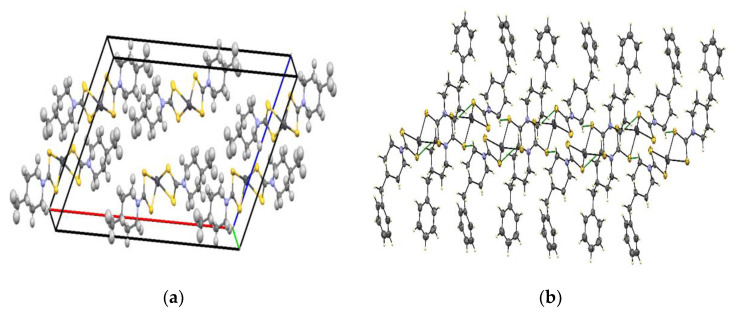
Crystal Packing diagrams of the lead(II)dithiocarbamate complexes. (**a**) [Pb(4-mpipdtc)2], (**b**) [Pb(4-bpipdtc)2].

The molecular structures of the Pb(II) dithiocarbamate compounds are shown in Figure 1 and Figure 2, packing diagrams in Figure 3, the crystallographic data are listed in Table 1, and relevant bond lengths and bond angles are presented in Table 2. [Pb(4-mpipdtc)_2_] is a mononuclear compound in a monoclinic crystal system with the C2/c space group. The monomeric unit consist of two molecules of methyl piperidinyl dithiocarbamato anions that are clustered on one side of the Pb(II) ion. The distorted tetrahedral coordination geometry indicates the existence of the stereochemical active 6*s*^2^ lone pair of electrons [20] to adopt the hemidirected geometry which is common for low coordinate lead(II) compounds [21]. The S2 atom forms a relatively strong coordination bond with the lead(II) ions with the intramolecular Pb1-S2 bond length of 2.7097 Å being shorter than that of Pb1-S1 (2.8831 Å), which is comparable to the bond lengths of other related lead(II) compounds in the literature [22]. The thioureide intermolecular C1—N2 bond length of 1.338(6) Å is associated with a C—N single bond (1.47 Å) and C=N double bond (1.28 Å) which indicates delocalization of the π-electrons. The S1—C1 and S2—C1 bond lengths are similar in length (1.721(4) Å) but shorter than the typical S—C (1.815 Å) and longer than C=S (1.671 Å), indicating a partial double bond feature. These results suggest a significant electron delocalization in the dithiocarbamate moiety. The geometry of the Pb(II) dithiocarbamate complexes could be described as a distorted tetrahedral geometry with a significantly acute S1—Pb—S2 bite angle of 64.02°.

[Pb(4-bpipdtc)_2_] crystallized in the monoclinic C2/n space group. The Pb(II) ion forms a distorted tetrahedral geometry with four sulphur atoms from the two dithiocarbamate anions, while the crystal structure core is comparable to other analogous compounds in the literature [23,24,25]. Two Pb—S have bond lengths of 2.8779(8) Å, which are longer than the other two Pb–S with bond length of 2.6614(7) Å. The S1—Pb1—S2 bite angle of 64.68° and dihedral angle of 84.74° are deviated from the perfect bond angles of a tetrahedral geometry. The crystal packing of [Pb(4-mpipdtc)_2_] (Figure 3a) shows six molecules of the compounds within the crystal packing arranged in group of three molecules that are parallel to each other. The crystal packing of [Pb(4-bpipdtc)_2_] consists of four molecules within the crystal packing in pairs that are parallel to each other. The compound is stabilized by inter C—H···S interactions (Figure 3b).

### 2.3. FTIR Spectroscopic Studies of the Lead(II) Dithiocarbamate

The v(–NCS_2_) frequency observed at 1468 cm^−1^ and 1469 cm^−1^ in the 4-methylpiperidine dithiocarbamate (*Mpipdtc)* and 4-benzylpiperidine dithiocarbamate *(Bpipdtc)* ligands shifted to 1429 cm^−1^ and 1478 cm^−1^ in [Pb(4-mpipdtc)_2_] and [Pb(4-Bpipdtct)_2_] complexes. This band lies between the C-N single bond and double bond characters and confirmed bonding of the dithiocarbamato anions to the Pb(II) ions [26,27]. The free ligands spectra showed single sharp bands due to ν(C−S) at 946 cm^−1^ for 4-methylpiperidine dithiocarbamate and 945 cm^−1^ for 4-benzylpiperidine dithiocarbamate. These bands shifted to 959 cm^−1^ and 961 cm^−1^ in their respective Pb(II) complexes. This indicates bidentate chelating bonding of the dithiocarbamato anions to the Pb(II) ions [28]. The electronic spectra of the free ligands reveal two bands at 261 and 280 nm due to the π → π* transition of the NCS and SCS thioureide moiety [29]. The Pb(II) complexes both exhibit bands at 265 nm due to charge transfer transitions, which is consistent with Pb(II) complexes [30].

### 2.4. Structural and Morphological Studies of the PbS Nanoparticles

#### 2.4.1. Powder X-ray Diffraction Studies of the PbS Nanoparticles

**Figure 4:**
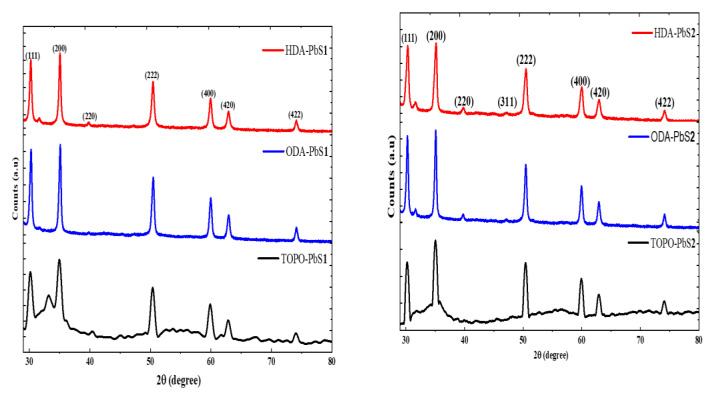
XRD diffraction patterns of PbS1 and PbS2 nanoparticles with different capping agents.

Structural identification of the PbS nanoparticles was performed with powder X-ray diffraction (pXRD). Figure 4 shows the pXRD patterns of HDA, ODA, and TOPO capped PbS nanoparticles. The peaks were indexed to the cubic rock salt phase of PbS that matches JCPDS file No. 5–0592 [31], which confirms the cubic crystal structures of PbS. The diffraction patterns of HDA-capped PbS nanoparticles showed prominent peaks at 2θ values of 30.29°, 35.10°, 50.49°, 60.01°, 62.98°, and 74.19°, which correlate with lattice planes (111), (200), (220), (311), (222), and (400) of the cubic rock salt phase of PbS (JCCD: 05-0592). The sharp peaks are ascribed to the crystalline nature of the PbS nanoparticles. ODA-capped PbS nanoparticles showed peaks at 30.30°, 35.12°, 50.48°, 60.59°, 62.96°, and 74.14° which can be indexed to (111), (200), (220), (311), (222), and (400) planes. The patterns are identical except for the absence a (220) peak in ODA-PbS1. This could be due to the change in the nanoparticle morphology. TOPO-capped nanoparticles showed diffraction patterns at 30.29°, 33.25°, 35.11°, 50.49°, 60.01°, 62.98°, and 74.16°and can be indexed to (111), (200), (220), (311), (222), and (400) lattice planes. The TOPO-capped PbS peaks are slightly widened compared to HDA- and ODA-capped PbS nanoparticles. The intensity of the (200) peak was greater than that of the (111) peak in all samples, implying a faster rate of growth on the 200 facets than on the 111 facets. The mean particle sizes of the PbS nanoparticles were computed from the diffraction planes (111), (200), and (222) using the Debye–Scherer formula [32]. The mean particle sizes were estimated as 25 nm for HDA-PbS1, 23 nm for HDA-PbS2, 22 nm for ODA-PbS1, 25nm for ODA-PbS2, and 26 and 35 nm for TOPO-PbS1 and TOPO-PbS2, respectively.

#### 2.4.2. HRTEM Micrographs of the PbS Nanoparticles

**Figure 5:**
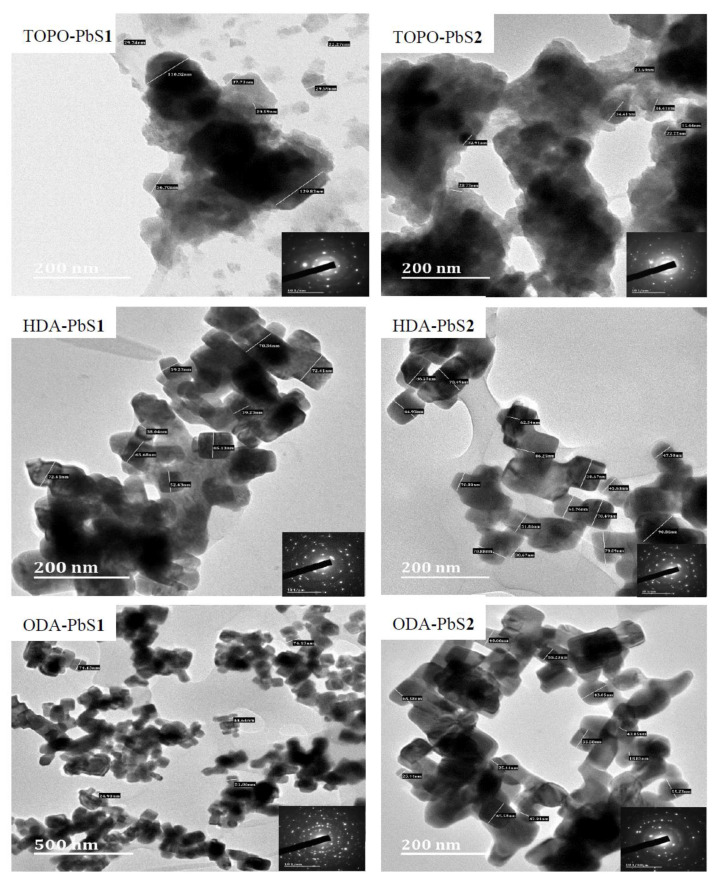
HRTEM images and SAED patterns of PbS nanoparticles.

**Figure 6:**
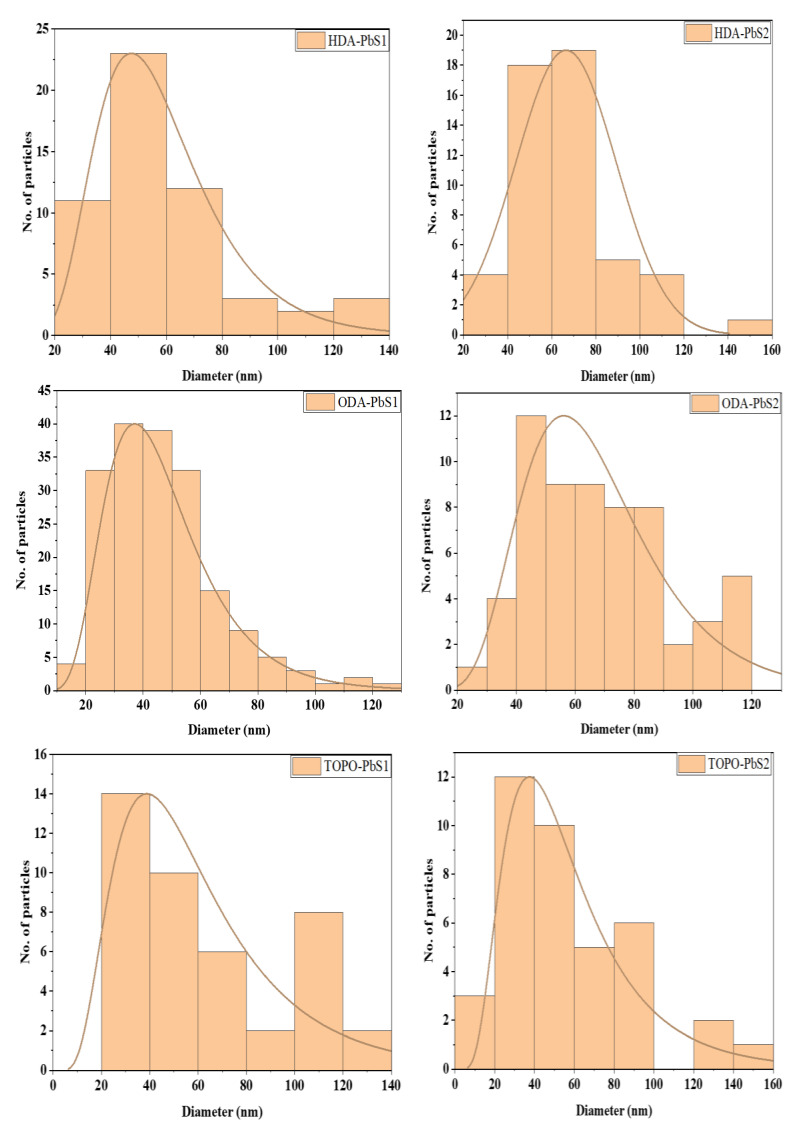
Particle size distributions of the as-prepared PbS nanoparticles.

HRTEM micrographs of the PbS nanoparticles (Figure 5) indicate that the HDA capped PbS nanoparticles exhibit agglomerated cubic-like structures with wide particle size ranges (Figure 6). The mean particle sizes are 58.88 nm for HDA-PbS**1** and 66.52 nm for HDA-PbS**2**. Octadecylamine capped PbS nanoparticles also show cubic-like shapes with mean particle sizes of 47.29 nm for ODA-PbS1 and 67.50 nm for ODA-PbS2, with some agglomeration that is more prominent in ODA-PbS1. PbS nanoparticles prepared from TOPO are densely packed, with TOPO-PbS1 showing wide particle sizes range of 22.27–129.83 nm with and average particle size of 61.31 nm while TOPO-PbS2 shows some spherically shaped particles with a mean particle size of 58.21 nm. Studies have shown that nanoparticles possess very high surface energy [33] and thus tend to agglomerate to reduce this surface energy [34]. Agglomeration in nanoparticles reduces the advantages of size effect, which could potentially affect any potential applications. Agglomerations could be caused by several factors, such as the ineffectiveness of the capping agent to passivate the surface of the as-prepared nanoparticles or too high a thermolysis temperature which causes rapid nucleation and growth, leading to the formation of agglomerated nanoparticles. The selected area electron diffraction (SAED) patterns depict concentric rings with bright ring spots for all the ODA and TOPO capped PbS nanoparticles, indicating that the PbS nanoparticles are of high crystallinity, while in both the HDA capped nanoparticles the bright spots are fewer, which could be ascribed to the agglomerated particles. The grain size of the particle increases because of agglomeration, and atomic mobility improves, resulting in greater crystallinity [35]. 

SEM images of HDA-capped PbS nanoparticles show an aggregated flake-like smooth surface morphology. EDX spectra of the materials showed significant peaks of Pb and S and the carbon is due to HDA while Au is attributed to coating with gold. For ODA capped nanoparticles, PbS2 revealed a solid smooth surface and PbS1 shows that the nanoparticles display a loosely agglomerated surface morphology with irregular spacing. It has been shown that reduced agglomeration of nanoparticles increases the accessible surface area on the material, which could lead to an increase in their photocatalytic efficiency [36]. Other studies have shown that if the surface morphology of nanoparticles is rough, it enhances the photocatalytic degradation efficiency of the material [37,38]. TOPO-PbS1 shows a flaky surface morphology while the TOPO-PbS2 SEM image reveals a closely packed spherical surface morphology. The EDX spectra show C and Au, P and O peaks due carbon tape, coating used, and the capping agent, respectively. The effect of capping with HDA, ODA, and TOPO is evidenced by the variations in the morphologies of the lead sulphide nanoparticles.

Fourier-transform infrared spectroscopy (FTIR) spectra of HDA, ODA, and TOPO capped lead sulphide nanoparticles show the asymmetric CH_3_ and CH_2_ vibrations in HDA-capped PbS nanoparticles spectra appeared at 2916 cm^−1^ and 2849 cm^−1^. The N–H stretching vibrations at 3243 cm^−1^ and the bending vibrations at 1630 cm^−1^ are the HDA capping agent distinctive peaks [39], which confirm that PbS nanoparticles are capped through contact of the –NH_2_ group on the HDA. Moreover, the omission of C–P stretching vibrations at 1170, 1010, and 1037 cm^−1^ indicates the absence of the tri-octylphosphine (TOP) on the surface of the PbS nanoparticles [40]. The spectra of PbS nanoparticles and pure ODA were also compared. The spectra exhibit few differences, indicating that the PbS nanoparticles interact with the capping agent. The three stretching frequencies found in the spectrum of ODA at 3326, 3248, and 3166 cm^−1^ are due to the v(N–H) stretching vibrations [41]. The variation in the intensities of the stretching vibrations in the PbS nanoparticles spectra indicate that the nanoparticles synthesized with different precursors led to the formation of PbS nanoparticles that interact with the capping agents differently. The TOPO-capped PbS spectra peaks were compared to the peaks of a clean TOPO spectrum. The CH_2_ frequencies are observed at 2915 and 2847 cm^−1^. With the exception of the P=O stretching frequency, the PbS nanoparticle stretching vibrations aligned in frequency with all of the TOPO peaks. The stretching vibrational band of P=O is observed at about 1145 cm^−1^ in the clean TOPO spectrum [42], whereas in the TOPO capped PbS nanoparticles spectra this peak shifted to 965 cm^−1^. The chemical interaction of TOPO molecules with PbS occurred via the P=O is responsible for the observed shift of this peak. The observed shift in P=O stretching vibrations is due to the capping of the nanoparticles by TOPO, which causes π-electron delocalization in P=O that effectively reduced the frequency of the P=O stretching mode, achieving as a result a lower absorption frequency, and red shift is observed [43].

### 2.5. Photocatalytic Studies

**Figure 7:**
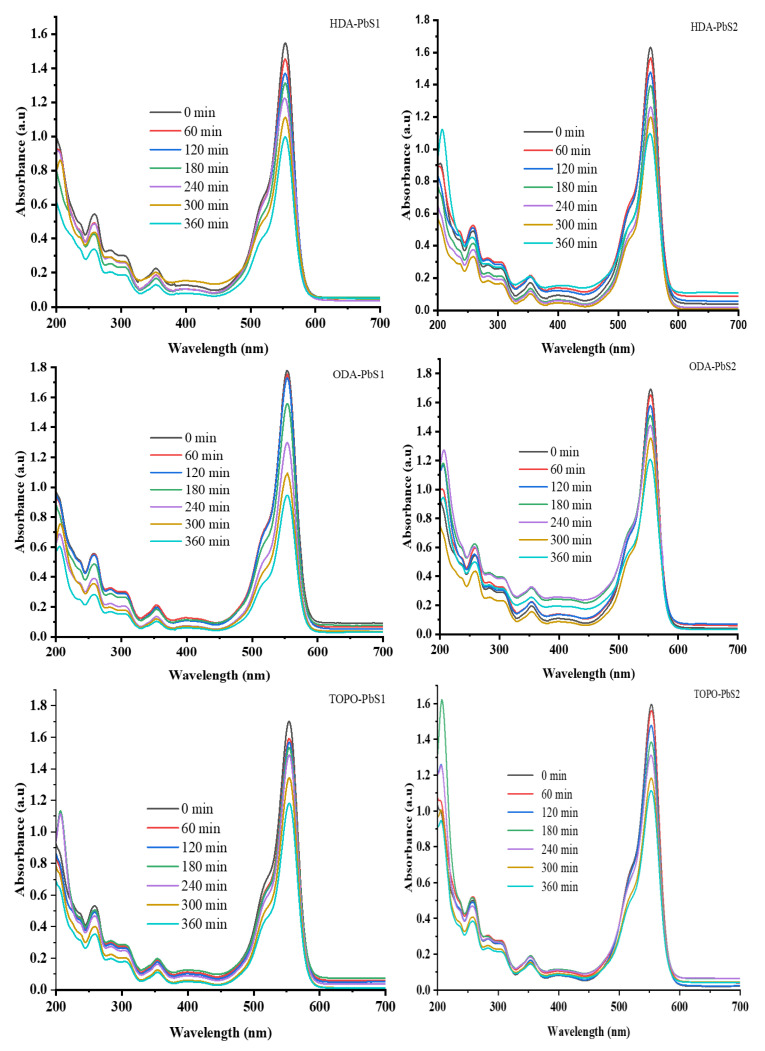
UV-Vis spectra of rhodamine B degradation by HDA-PbS, ODA-PbS and TOPO-PbS nano photocatalysts.

**Figure 8:**
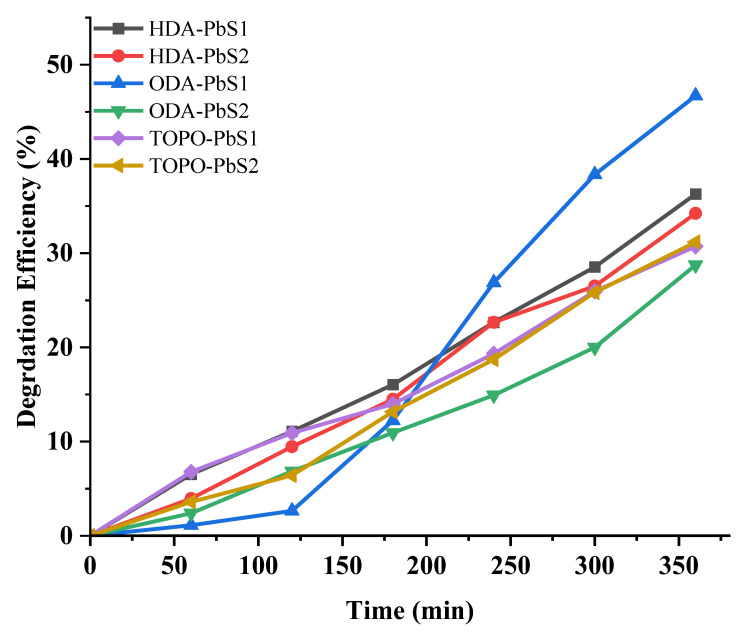
Degradation efficiency plot of rhodamine B using HDA-PbS, ODA-PbS and TOPO-PbS as photocatalysts.

The organic dye rhodamine B was used as a model in the photocatalytic evaluation of HDA, ODA, and TOPO capped PbS nanoparticles. The absorption spectra in Figure 7 show a time dependent reduction in absorption maxima at 553 nm, which was used to calculate degradation efficiency using a previously reported methodology [44,45]. The findings show that after 360 min, only about 45% of the dye was degraded (Figure 8). The order of photocatalytic degradation of rhodamine B by the PbS nanoparticles is as follows: ODA-PbS2 > TOPO-PbS1 > TOPO-PbS2 > HDA-PbS2 > HDA-PbS1 > ODA-PbS1. It has been reported that pure PbS nanoparticles have poor degradation efficiency due to poor separation that causes a fast recombination of electron-hole pairs [46,47]. ODA-PbS1 exhibited the highest photodegradation efficiency of rhodamine B dye. The photocatalytic degradation of bromothymol blue by hexadecylamine-PbS nanoparticles showed the highest degradation efficiency of 66% [29], while oleylamine-PbS degraded 73.89% of methylene blue [45]. Oleic acid-PbS nanoparticles prepared from bis(phenylpiperazine dithiocarbamato)lead(II) degraded 50.58% of rhodamine B [46]. The highest photocatalytic efficiency of rhodamine B obtained in this study is comparable to that of PbS nanoparticles prepared from another single precursor.

**Figure 9:**
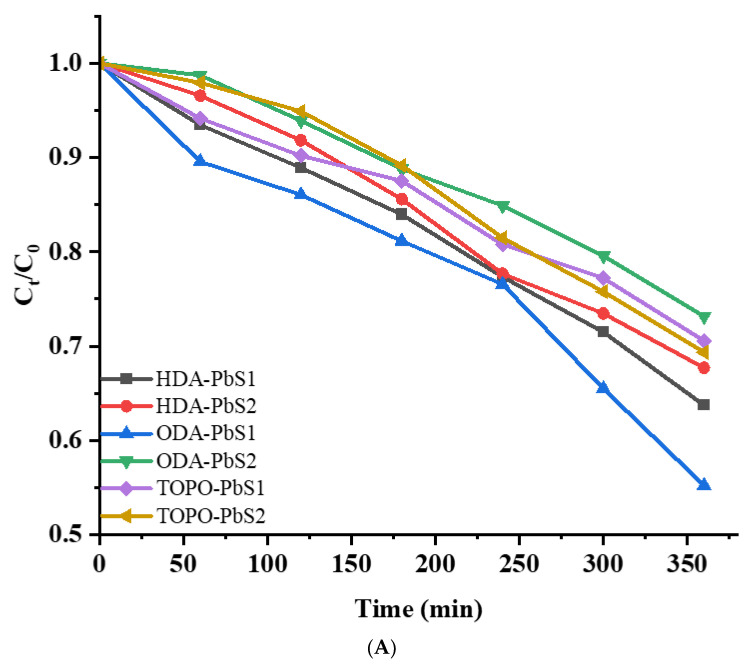

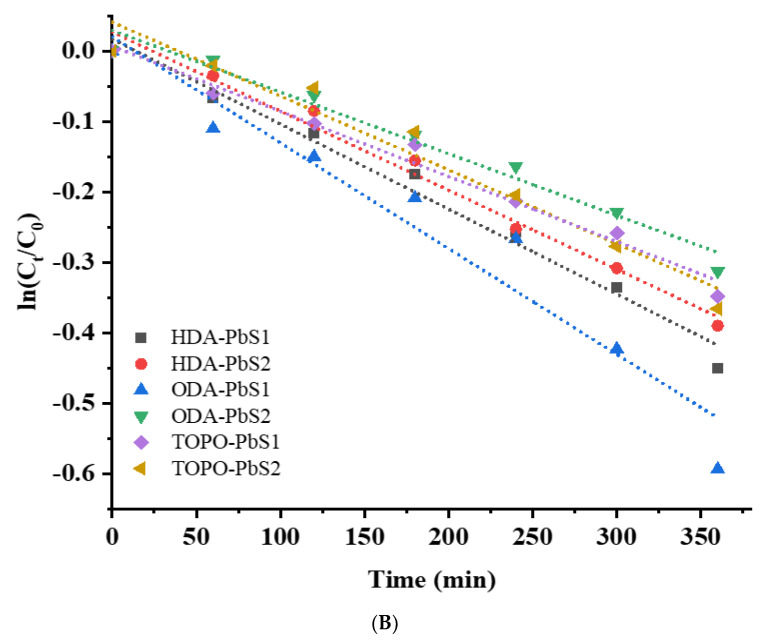
(**A**) Effect of time on Rhodamine B degradation (**B**) kinetic plot of rhodamine B using HDA-PbS, ODA-PbS and TOPO-PbS as photocatalysts.

A pseudo-first-order kinetics approach was adopted using the Langmuir–Hinshelwood kinetic model to investigate the rate of rhodamine B degradation in the presence of PbS nanoparticles over time (Figure 9) [48]. The percentage degradation, rate constants, and correlation coefficients (R^2^) are shown in Figure 8 and presented in Table 3. The high R^2^ (>0.90) values show that the photocatalytic degradation of rhodamine B fits the pseudo-first-order kinetic equation [49], in agreement with the photo degradation efficiency curve. Despite similar degradation efficiencies, the photodecomposition rates of rhodamine B dye by the as-prepared PbS nano photocatalysts are different.

**Figure 10:**
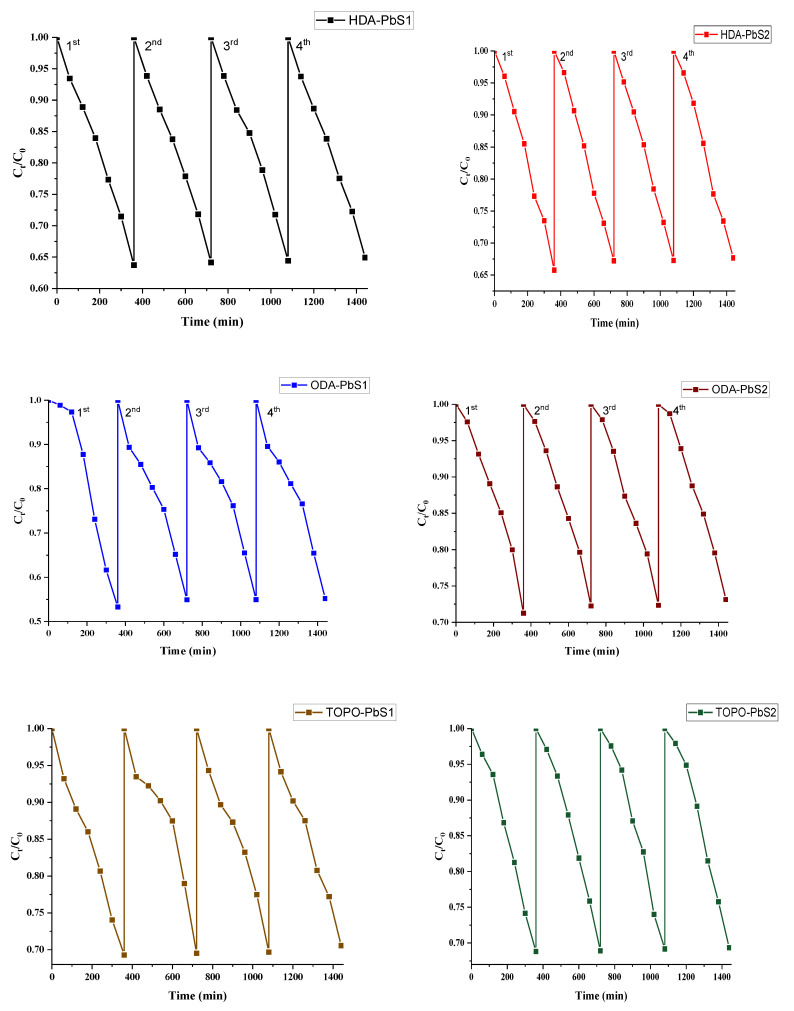
The reusability of PbS photocatalysts.

**Figure 11:**
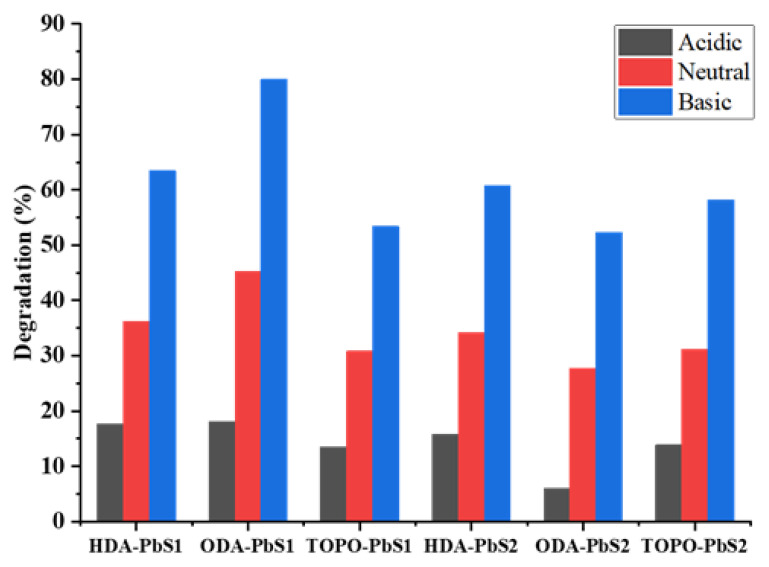
Effect of pH on photocatalytic degradation of rhodamine B by HDA-PbS, ODA-PbS and TOPO-PbS nanoparticles.

The photostability and reusability of the as-prepared PbS nanoparticles were tested. After each photocatalytic cycle, the photocatalyst was centrifuged, washed with methanol, and dried. The photocatalyst was reused four times under the same conditions, the results shows that the percentage degradation efficiency decreased slightly by about 0.5–1.93% from 1st cycles up to the 4th cycle (Figure 10 and Figure S10, Table ST1). The TOPO-PbS2 is the most photostable among the as-prepared PbS nanoparticles. The effect of the solution pH is shown in Figure 11. Experiments were conducted at a fixed concentration at pH 4, 6.5, and 10. Studies have shown that any change in the pH of the medium can affect the dyes and catalyst charges as well as the rate of adsorption on the catalyst active sites [50]. It isNo evident that the efficiency of RhB decomposition improves at higher pH. The dye exists in the cationic form (RhB+) in acidic medium [51]. As a result, electrostatic repulsion between RhB and the catalysts may arise, lowering the degradation efficiency. RhB+ is deprotonated at higher pH values, forming the zwitter ion. Furthermore, a basic medium may lead to the formation of hydroxyl radicals, which leads to degradation via the •OH radical oxidation pathway. These factors can aid the breakdown of RhB and reaction intermediates.

## 3. Experimental

### 3.1. Characterization Techniques

All reagents were bought from Merck (Burlington, Massachusetts, United States) and used without further purifications. FTIR spectra were measured between 4000 and 650 cm^−1^ on an Agilent Technologies Cary 630 FTIR instrument (Agilent Technology, Santa Clara, California USA). The ligands and bis(4-methylpiperidine-1-carbodithioato)-lead(II) and bis(4-benzylpiperidine-1-carbodithioato)-lead(II) were characterized using ^1^H-NMR and ^13^C-NMR on a 400 MHz Bruker Avance III NMR spectrometer (Billerica, Massachusetts, USA). with TMS (tetramethylsilane) as an internal standard. A Perkin Elmer (Waltham, Massachusetts, United States) Lambda 25 UV-Vis spectrometer was used for absorption analysis. On a Bruker D8 (Billerica, MA, USA), advance diffractometer, powder X-ray diffraction patterns were obtained using monochromatic CuKa radiation (λ = 1.5418). Perkin Elmer (Waltham, MA, USA) LS 45 fluorescence was used to gather photoluminescence spectra. A Joel 1400 Transmission Electron Microscope (Akishima, Tokyo, Japan) was used to take TEM pictures. A ZEISS EVO LS 15 Scanning Electron Microscope (Oberkochen, Germany) was used to collect SEM and EDS pictures.

### 3.2. Synthesis of Sodium Salt 4-Methylpiperidine Dithiocarbamate Ligand

An aqueous solution of 0.05 mol (2.00 g) sodium hydroxide was reacted with 0.05 mol (4.9585 g) of 4-methylpiperidine, after which 0.05 mol (3.00 mL) previously cooled carbon disulphide was added and agitated for 4 h on ice kept at 0–4 °C. The product was filtered and rinsed with diethyl ether. Yield: 6.9112 g, 70.15%. Mp: 139.6 °C–143.3 °C. FTIR υ(cm^−1^): 1468 (C–N), 945 (C–S). ^1^H-NMR (D_2_O): 0.91 (3H, d, CH_3_), 1.69–1.73 (^1^H, s, CH), 1.11–1.21 (4H, s, CH_2_), 3.11–3.18 (4H, s, CH_2_). ^13^C-NMR (D_2_O): 20.5 (CH_3_), 30.1 (CH), 33.6 (CH_2_), 52.2 (CH_2_), 205.5 (CS_2_).

### 3.3. Synthesis of Sodium Salt 4-Benzylpiperidine Dithiocarbamate Ligand

Carbon disulfide 0.03 mol (1.80 mL) was slowly added to an equimolar mixture of sodium hydroxide 0.03 mol (1.20 g) and 4-benzylpiperidine 0.03 mol (5.2581 g), which was chilled in an ice bath. The reaction was stirred for 4 h. The resulting white precipitate, was collected, washed with diethyl ether, and suction dried. Yield: 3.0452 g, 37.17%. Mp: 128.2 °C–131.4 °C. Selected FTIR υ(cm^−1^): 1417 (C–N), 945 (C–S). ^1^H-NMR (D_2_O): 1.19–1.31 (2H, m, CH_2_), 1.67–1.70 (2H, d, CH_2_), 3.05–3.12 (2H, s, CH_2_), 5.35–5.38 (2H, d, CH_2_), 1.86–1.97 (1H, m, CH), 2.56–2.58 (2H, d, CH_2_), 7.22–7.36 (5H, m, C_6_H_5_). ^13^C-NMR (D_2_O): 126.1–129.2 (C_6_H_5_), 141.4 (C-C_6_H_5_), 41.7 (CH_2_), 37.8 (CH), 31.7 (CH_2_), 52.0 (CH_2_), 205.9 (CS_2_).

### 3.4. Synthesis of Pb(II) Dithiocarbamate Complexes 

Bis(4-methylpiperidine-1-carbodithioato)-lead(II) and bis(4-benzylpiperidine-1-carbodithioato)-lead(II) were synthesized by adding the ligands sodium salt 4-methylpiperidine dithiocarbamate or sodium salt 4-benzylpiperidine dithiocarbamate in distilled water dropwise to the solution of lead nitrate. The precipitate formed instantly, and the reaction was completed by stirring the mixture for 1 h, before being filtered, washed, and dried in vacuum.

**[Pb(4-Mpip)_2_]:** Yield: 1.5731 g, 91.58%. Mp: 261.5–263.4 °C. ^1^H-NMR (DMSO): 0.90 (6H, d, CH_3_), 1.71–1.74 (2H, t, CH), 1.03–1.12 (8H, q, CH_2_), 2.99–3.05 (8H, q, CH_2_). FTIR υ(cm^−1^): 958 (CS_2_) and 1429 (N-CS_2_). TOF MS ES^+^ (m/z): 552.93.

**[Pb(4-Bpip)_2_]:** Yield: 1.6316 g, 92.16%. Mp: 211.2 °C–214.6 °C. Selected FTIR υ(cm^−1^): 1478 (C–N), 961 (C–S). ^1^H-NMR (D_2_O): 1.19–1.31 (2H, m, CH_2_), 1.67–1.70 (2H, d, CH_2_), 3.05–3.12 (2H, s, CH_2_), 5.35–5.38 (2H, d, CH_2_), 1.86–1.97 (1H, m, CH), 2.56–2.58 (2H, d, CH_2_), 7.22–7.36 (5H, m, C_6_H_5_).

### 3.5. X-ray Crystallography

Slow evaporation of chloroform solution yielded single crystals of [Pb(4-Mpip)_2_] and [Pb(4-Bpip)_2_] complexes. Appropriate crystals were chosen and mounted on a MITIGEN holder in paratone oil fitted on a Bruker APEX-II CCD diffractometer (Billerica, MA, USA). During analysis, the crystals were held at 102.54 K. Using Olex2 [52], structure solution program SHELXS was used to analyse the molecular structure [53] using direct methods and simplified with the refinement package SHELXL [54] by least squares minimization. 

### 3.6. Preparation of PbS Nanoparticles

Briefly, 0.25 g of each Pb(II) dithiocarbamate complex was dispersed in 1 mL of trioctylphosphine (TOP). The resulting solution was introduced into 4 g of hot octadecylamine (ODA) in a three-necked round bottom flask at 180 °C under an inert atmosphere and constant magnetic stirring. After a 12–18 °C temperature drop, the reaction was allowed to stabilize at the desired temperature, then agitated for 1 h, and cooled to 70 °C, after which methanol was added to precipitate the nanoparticles. The products were then centrifuged at 3500 rpm for 30 min, decanted, and washed numerous times to remove excess ODA. The same procedure was repeated with hexadecylamine (HDA) and trioctylphosphine oxide (TOPO) to investigate the influence of capping agents.

### 3.7. Photodegradation of Rhodamine B Dye

The degradation efficiency of the PbS nanoparticles was investigated by observing the degradation of rhodamine B (10 mg/L) under an 80 W mercury lamp. In a 50 mL dye solution, 50 mg of PbS nanoparticles were used as catalysts. To achieve the adsorption balance on the catalyst’s surface, the solution was sonicated for 30 min and then agitated for 60 min in the dark. The solutions were subjected to a high-pressure visible light source for 360 min. The samples were then centrifuged to remove the nanoparticles, and the dye solution was taken for absorption analysis with a UV-Vis spectrophotometer at 60-min intervals.

## 4. Conclusions

Bis(4-methylpiperidine-1-carbodithioato)lead(II) and bis(4-benzylpiperidine-1-carbodithioato)lead(II) were prepared and analysed with spectroscopic methods and X-ray crystallography. The compounds’ structures showed monomeric lead(II) complexes with lead(II) ions bonding to two dithiocarbamato anions to form a distorted tetrahedral geometry. The compounds were thermolyzed at 180 ℃ to prepare hexadecylamine (HDA), octadecylamine (ODA), and trioctylphosphine oxide (TOPO) capped lead sulphide (PbS) nanoparticles. pXRD showed that the nanoparticles are in the cubic rock salt phase of PbS. The photo catalytic activity of the as-prepared PbS nanoparticles was examined using rhodamine B dye in aqueous solution. The study shows that after 360 min, only about 45% of the dye was degraded. The photodegradation efficiency of the PbS nanoparticles are in the order ODA-PbS2 > TOPO-PbS1 > TOPO-PbS2 > HDA-PbS2 > HDA-PbS1 > ODA-PbS1. The photostability and reusability of the synthesized PbS nanoparticles were tested. Recyclability studies show that the PbS nanoparticles are reusable and relatively photostable, with a slight decrease in the degradation efficiency of about 0.51–1.93% and with TOPO-PbS2 being the most photostable. The study of the effect of pH shows that the photo degradation efficiency of rhodamine B by the PbS nanoparticles improves at pH higher than pH.5.

## Figures and Tables

**Table 1:** Crystallographic data and structure refinements for the complexes.

Compound	[Pb(4-Mpipdtc)_2_]	[Pb(4-Bpipdtc)_2_]
SFormula	C_14_H_24_N_2_PbS_4_	C_26_H_32_N_2_PbS_4_
*D_calc_.*/g cm^−3^	1.951	1.724
μ/mm^−1^	21.417	15.012
Formula Weight	555.78	707.96
Size/mm^3^	0.505 × 0.190 × 0.055	0.335 × 0.270 × 0.120
*T*/K	102(2)	102(2)
Crystal System	Monoclinic	Monoclinic
Space Group	C2/c	C2/n
*a*/Å	29.5086(10)	25.5177(17)
*b*/Å	4.6830(2)	9.1660(5)
*c*/Å	15.6621(5)	12.0217(7)
α/^°^	90	90
β/^°^	119.0530(10)	104.015(4)
γ/^°^	90	90
V/Å^3^	1891.99(12)	2728.1(3)
*Z*	4	4
*Z’*		
*Θ_min_*/^°^	3.427	0.335
*Θ_max_*/^°^	72.650	72.234
Measured Refl.	16061	26753
Independent Refl.	1838	2679
Reflections Used	100	123
*R_int_*	0.0547	0.0461
Parameters	97	150
Largest Peak	0.452	0.399
Deepest Hole	−2.382	−1.393
GooF	1.108	1.138
*wR*_2_ (all data)	0.0870	0.0563
*wR*_2_	0.0856	0.561
*R*_1_ (all data)	0.0349	0.0229
*R*_1_	0.0338	0.0227

**Table 2:** Selected bond lengths and bond angles for the Pb(II) complexes.

[Pb(4-Mpipdtc)_2_]		[Pb(4-Bpipdtc)_2_]	
Bond	Length(Å)	Bonds	Length(Å)
Pb1—S2	2.7097(10)	Pb1—S1	2.6614(7)
Pb1—S2	2.7097(10)	Pb1—S1	2.6614(7)
Pb1—S1	2.8831(13)	Pb1—S2	2.8778(8)
Pb1—S1	2.8831(13)	Pb1—S2	2.8779(8)
S2—C1	1.736(4)	S2—C1	1.710(3)
S1—C1	1.705(4)	S1—C1	1.738(3)
C1—N2	1.338(6)	N1—C1	1.323(4)
**Bond**	**Angle(°)**	**Bonds**	**Angle(°)**
S2—Pb1—S2	97.63(5)	S1—Pb1—S1	91.56(3)
S2—Pb1—S1	64.02(3)	S1—Pb1—S2	84.75(2)
S2^1^—Pb1—S1	83.57(3)	S1^1^—Pb1—S2	64.68(2)
S2—Pb1—S1	83.57(3)	S1—Pb1—S2	64.68(2)
S2^1^—Pb1—S1	64.02(3)	S1^1^—Pb1—S2	84.76(2)
S1—Pb1—S1	130.63(5)	S2^1^—Pb1—S2	136.31(3)
C1—S2—Pb1	90.67(14)	C1—S1—Pb1	91.46(9)
C1—S1—Pb1	85.60(15)	C1—S2—Pb1	84.94(9)

**Table 3:** Percentage photo catalytic degradation spectra of rhodamine B, rate constants of photodegradation and its corresponding correlation coefficient.

Compound	Degradation (%)	Rate Constant (min^−1^)	R^2^
HDA-PbS**1**	36.18	0.00121	0.9816
HDA-PbS**2**	34.22	0.00116	0.9817
ODA-PbS**1**	45.28	0.01981	0.9413
ODA-PbS**2**	27.75	8.85 × 10^−^^4^	0.9695
TOPO-PbS**1**	30.81	9.53 × 10^−^^4^	0.9805
TOPO-PbS**2**	31.17.	0.00106	0.9731

## Data Availability

CCDC 2077062 and CCDC 2079500 contain supplementary crystallographic data can be obtained from the Cambridge Crystallographic Data Centre via www.ccdc.cam.ac.uk/data_request/cif or from The Director, CCDC, 12 Union Road, Cambridge, CB2 1EZ, UK (Fax: + 44-1223-336-033; or email: deposit@ccdc.cam.ac.uk).

## Supplementary Materials

The following are available online. Figure S1: Proton NMR spectrum of 4-methylpiperidine dithiocarbamate, Figure S2: Proton NMR spectrum of 4-benzylpiperidine dithiocarbamate, Figure S3: Proton NMR spectrum of bis(4-methylpiperidine-1-carbodithioato)-lead(II), Figure S4: Proton NMR spectrum of bis(4-benzylpiperidine-1-carbodithioato)-lead(II), Figure S5: FTIR spectrum of bis(4-methylpiperidine-1-carbodithioato)-lead(II), Figure S6: FTIR spectrum of bis(4-benzylpiperidine-1-carbodithioato)-lead(II), Figure S7: ToF mass spectrum of bis(4-methylpiperidine-1-carbodithioato)-lead(II), Figure S8: ToF mass spectrum of bis(4-benzylpiperidine-1-carbodithioato)-lead(II), Figure S9. FTIR spectra of (HDA, ODA, and TOPO) capped as-prepared PbS nanoparticles, Figure S10. The reusability of PbS photocatalysts, Table ST1: Percentage (%) degradation for recyclability studies.

## References

[1] Angeloski A., Gentle A.R., Scott J.A., Cortie M.B., Hook J.M., Westerhausen M.T., Bhadbhade M., Baker A.T., McDonagh A.M. (2018). From lead(II) dithiocarbamate precursors to a fast response PbS positive temperature coefficient thermistor. Inorg. Chem..

[2] Bederak D., Dirin D.N., Sukharevska N., Momand J., Kovalenko M.V., Loi M.A. (2021). S-Rich PbS quantum dots: A promising p-type material for optoelectronic devices. Chem. Mater..

[3] Saraidarov T., Reisfeld R., Sashchiuk A., Lifshitz E. (2007). Synthesis and characterization of PbS nanorods and nanowires. Phys. E Low-Dimens. Syst. Nanostruct..

[4] Akkerman Q.A., Martín-García B., Buha J., Almeida G., Toso S., Marras S., Bonaccorso F., Petralanda U., Infante I., Manna L. (2019). Ultrathin orthorhombic PbS nanosheets. Chem. Mater..

[5] Kord M., Hedayati K., Farhadi M. (2017). Green synthesis and characterization of flower-like PbS and metal-doped nanostructures via hydrothermal method. Main Group Met. Chem..

[6] Saah S.A., Boadi N.O., Adu-Poku D., Wilkins C. (2019). Lead ethyl dithiocarbamates: Efficient single-source precursors to PbS nanocubes. Roy. Soc. Open Sci..

[7] Song C., Sun M., Yin Y., Xiao J., Dong W., Li C., Zhang L. (2016). Synthesis of star-shaped lead sulfide (PbS) nanomaterials and theirs gas-sensing properties. Mater. Res..

[8] Wang D., Yu D., Shao M., Liu J., Yu W., Qian Y. (2003). Dendritic growth of PbS crystals with different morphologies. J. Cryst. Growth.

[9] Li F., Huang X., Kong T., Liu X., Qin Q., Li Z. (2009). Synthesis and characterization of PbS crystals via a solvothermal route. J. Alloy. Compd..

[10] Bozkurt P.A., Derkuş B., Emregül K.C., Canel M. (2015). Sonochemical synthesis and characterisation of lead sulfide nanoparticles using different capping agents. J. Chem. Res..

[11] Kumar D., Agarwal G., Tripathi B., Vyas D., Kulshrestha V. (2009). Characterization of PbS nanoparticles synthesized by chemical bath deposition. J. Alloy. Compd..

[12] Roffey A., Hollingsworth N., Hogarth G. (2019). Synthesis of ternary sulfide nanomaterials using dithiocarbamate complexes as single source precursors. Nanoscale Adv..

[13] Garcia-Gutierrez D.F., Hernandez-Casillas L.P., Cappellari M.V., Fungo F., Martínez-Guerra E., García-Gutiérrez D.I. (2018). Influence of the Capping Ligand on the Band Gap and Electronic Levels of PbS Nanoparticles through Surface Atomistic Arrangement Determination. ACS Omega.

[14] Niu Z., Li Y. (2014). Removal and Utilization of Capping Agents in Nanocatalysis. Chem. Mater..

[15] Olenin A.Y., Krutyakov Y.A., Kudrinskii A.A., Lisichkin G.V. (2008). Formation of surface layers on silver nanoparticles in aqueous and water-organic media. Colloid J..

[16] Jadhav S.A., Brunella V., Scalarone D. (2015). Polymerizable ligands as stabilizers for nanoparticles. Part. Part. Syst. Charact..

[17] Mphahlele L.L.R., Ajibade P.A. (2020). CdS quantum dots as photocatalyst for methylene blue and methyl red degradation and its electrochemical properties. Int. J. Electrochem. Sci..

[18] Ajibade P.A., Solomane N. (2020). Synthesis and crystal structure of bis(thiomorpholinyldithiocarbamato) Zn(II): Structural, optical and photocatalytic studies of ZnS nanoparticles from the complex. J. Coord. Chem..

[19] Oluwalana A.E., Ajibade P.A. (2019). Effect of temperature and capping agents on structural and optical properties of tin sulphide nanocrystals. J. Nanotech..

[20] Claudio E.S., Godwin H.A., Magyar J.S. (2002). Fundamental Coordination Chemistry, Environmental Chemistry, and Biochemistry of Lead(II). Progress in Inorganic Chemistry.

[21] Wu X.-S., Tang Y.-R., Liu J.-L., Wang L., Ren X.-M. (2019). Comprehensively understanding the steric hindrance effect on the coordination sphere of Pb2+ ions and photophysical nature of two luminescent Pb(II)-coordination polymers. Dalton Trans..

[22] Breza M., Bučinský L., Šoralová S., Biskupič S. (2010). On the origin of the hemidirected geometry of tetracoordinated lead(II) compounds. Chem. Phys..

[23] Iwasaki H., Hagihara H. (1972). The crystal structure of lead(II) diethyldithiocarbamate. Acta Crystallogr. B.

[24] Ito M., Iwasaki H. (1980). Structure of lead(II) N,N-diisopropyldithiocarbamate [bis(N,N-diisopropyldithiocarbamato)lead(II)]. Acta Crystallogr. B.

[25] Akhtar J., Malik M.A., O’Brien P., Helliwell M. (2010). Controlled synthesis of PbS nanoparticles and the deposition of thin films by Aerosol-Assisted Chemical Vapour Deposition (AACVD). J. Mater. Chem..

[26] Afzaal M., Ellwood K., Pickett N.L., O’Brien P., Raftery J., Waters J. (2004). Growth of lead chalcogenide thin films using single-source precursors. J. Mater. Chem..

[27] Ajibade P.A., Oluwalana A.E., Andrew F.P. (2020). Morphological studies, photocatalytic activity, and electrochemistry of platinum disulfide nanoparticles from bis(morpholinyl-4-carbodithioato)-platinum(II). ACS Omega.

[28] Sathiyaraj E., Thirumaran S. (2012). Synthesis and spectral studies on Pb(II) dithiocarbamate complexes containing benzyl and furfuryl groups and their use as precursors for PbS nanoparticles. Spectrochim. Acta A.

[29] Oluwalana A.E., Ajibade P.A. (2020). Synthesis and crystal structures of Pb(II) dithiocarbamates complexes: Precursors for PbS nanophotocatalyst. J. Sulfur Chem..

[30] Ajibade P., Mbese J., Omondi B. (2016). Group 12 dithiocarbamate complexes: Synthesis, characterization and X-ray crystal structures of Zn(II) and Hg(II) complexes and their use as precursors for metal sulfide nanoparticles. Synth. React. Inorg. Met..

[31] Singh H.L., Singh J.B., Bhanuka S. (2016). Synthesis, spectral, DFT, and antimicrobial studies of tin(II) and lead(II) complexes with semicarbazone and thiosemicarbazones derived from (2-hydroxyphenyl)(pyrrolidin-1-yl)methanone. J. Coord. Chem..

[32] Meng W., Yuan W., Wu Z., Wang X., Xu W., Wang L., Zhang Q., Zhang C., Wang J., Song Q. (2019). Mechanochemical synthesis of lead sulfide (PbS) nanocrystals from lead oxide. Powder Technol..

[33] Mamiyev Z., Balayeva N. (2015). Preparation and optical studies of PbS nanoparticles. Opt. Mater..

[34] Nanda K.K., Maisels A., Kruis F.E., Fissan H., Stappert S. (2003). Higher surface energy of free nanoparticles. Phys. Rev. Lett..

[35] Tang E., Cheng G., Ma X., Pang X., Zhao Q. (2006). Surface modification of zinc oxide nanoparticle by PMAA and its dispersion in aqueous system. Appl. Surf. Sci..

[36] Gupta D., Chauhan R., Kumar N., Singh V., Srivastava V.C., Mohanty P., Mandal T.K. (2020). Enhancing photocatalytic degradation of quinoline by ZnO:TiO_2_ mixed oxide: Optimization of operating parameters and mechanistic study. J. Environ. Manag..

[37] Arularasu M. (2019). Effect of organic capping agents on the optical and photocatalytic activity of mesoporous TiO_2_ nanoparticles by sol–gel method. JSN Appl. Sci..

[38] Gao H., Lu B., Liu F., Liu Y., Zhao X. (2012). Photocatalytical properties and theoretical analysis of N, Cd-Codoped TiO_2_ synthesized by thermal decomposition method. Inter. J. Photoenergy.

[39] Estrada-Flores S., Martínez-Luévanos A., Perez-Berumen C.M., García-Cerda L.A., Flores-Guia T.E. (2020). Relationship between morphology, porosity, and the photocatalytic activity of TiO_2_ obtained by sol–gel method assisted with ionic and nonionic surfactants. Bol. Soc. Esp. Ceram. V..

[40] Ajibade P.A., Mbuyazi T.B., Oluwalana A.E. (2021). Lead sulphide nanoparticles as photocatalyst for the degradation of methylene blue: Effects of ph, time, adsorption kinetics and recyclability studies. J. Inorg. Organomet. Polym. Mater..

[41] Chen S., Zhang X., Zhang Q., Tan W. (2009). Trioctylphosphine as both solvent and stabilizer to synthesize CdS nanorods. Nanoscale Res. Lett..

[42] Ahmad D.N., Chieng B.W., Ibrahim N.A., Talib Z.A., Muhamad E.N., Abidin Z.Z. (2017). Functionalizing graphene oxide with alkylamine by gamma-ray irradiation method. Nanomaterials.

[43] Ma J. (2011). Preparation and characterization of ZrO_2_ nanoparticles capped by trioctylphosphine oxide (TOPO). J. Wuhan Univ. Technol..

[44] Ajibade P.A., Oluwalana A.E. (2020). Structural, optical, photocatalytic and electrochemical studies of PbS nanoparticles. J. Nano Res..

[45] Oluwalana A.E., Ajibade P.A. (2020). Structural, optical, and photocatalytic studies of hexadecylamine-capped lead sulfide nanoparticles. Inter. J. Industr. Chem..

[46] Oluwalana A.E., Ajibade P.A. (2021). Structural, optical, and photocatalytic studies of oleylamine capped PbS nanoparticles. Opt. Quant. Electr..

[47] Suganya M., Balu A.R., Balamurugan S., Srivind J., Narasimman V., Manjula N., Rajashree C., Nagarethinam V.S. (2018). Photoconductive, photocatalytic and antifungal properties of PbS:Mo nanoparticles synthesized via precipitation method. Surf. Inter..

[48] Chiu Y.-H., Chang T.-F.M., Chen C.-Y., Sone M., Hsu Y.-J. (2019). Mechanistic insights into photodegradation of organic dyes using heterostructure photocatalysts. Catalysts.

[49] Solomane N., Ajibade P.A. (2021). Synthesis and crystal structure of bis(thiomorpholinyldithiocarbamato)Cu(II) complex and its use as precursor for CuS nanoparticles photocatalyst for the degradation of organic dyes. J. Sulfur Chem..

[50] Antoniou M., Dionysiou D. (2007). Application of immobilized titanium dioxide photocatalysts for the degradation of creatinine and phenol, model organic contaminants found in NASA’s spacecrafts wastewater streams. Catal. Today.

[51] Hariprasad N., Anju S.G., Yesodharan E.P. (2013). Sunlight induced removal of rhodamine B from water through semiconductor photocatalysis: Effects of adsorption, reaction conditions and additives. Res. J. Mater. Sci..

[52] Dolomanov O.V., Bourhis L.J., Gildea R.J., Howard J.A.K., Puschmann H. (2009). OLEX2: A complete structure solution, refinement and analysis program. J. Appl. Crystallogr..

[53] Sheldrick G. (2008). A short history of SHELX. Acta Crystallogr. A.

[54] Sheldrick G. (2015). Crystal structure refinement with SHELXL. Acta Crystallogr. C.

