# Supplementary material for: Bis(4-methylpiperidine-1-carbodithioato)-lead(II) and Bis(4-benzylpiperidine-1-carbodithioato)-lead(II) as Precursors for Lead Sulphide Nano Photocatalysts for the Degradation of Rhodamine B
Source: Molecules. 2021 Nov 29;26(23):7251. doi: 10.3390/molecules26237251 (PMC8658782; doi:10.3390/molecules26237251)
Supplement: Supplementary file 1 [file molecules-26-07251-s001.zip › molecules-1433509-supplementary.pdf]

Synthesis, Structural, Optical, and Photocatalytic Studies of PbS Nanocrystals

Thandi B. Mbuyazi and Peter A. Ajibade *

School of Chemistry and Physics, University of KwaZulu-Natal, Private Bag X01, Scottsville, Pietermaritzburg 3209, South Africa; 216006335@stu.ukzn.ac.za

* Correspondence: ajibadep@ukzn.ac.za

Supplementary Materials

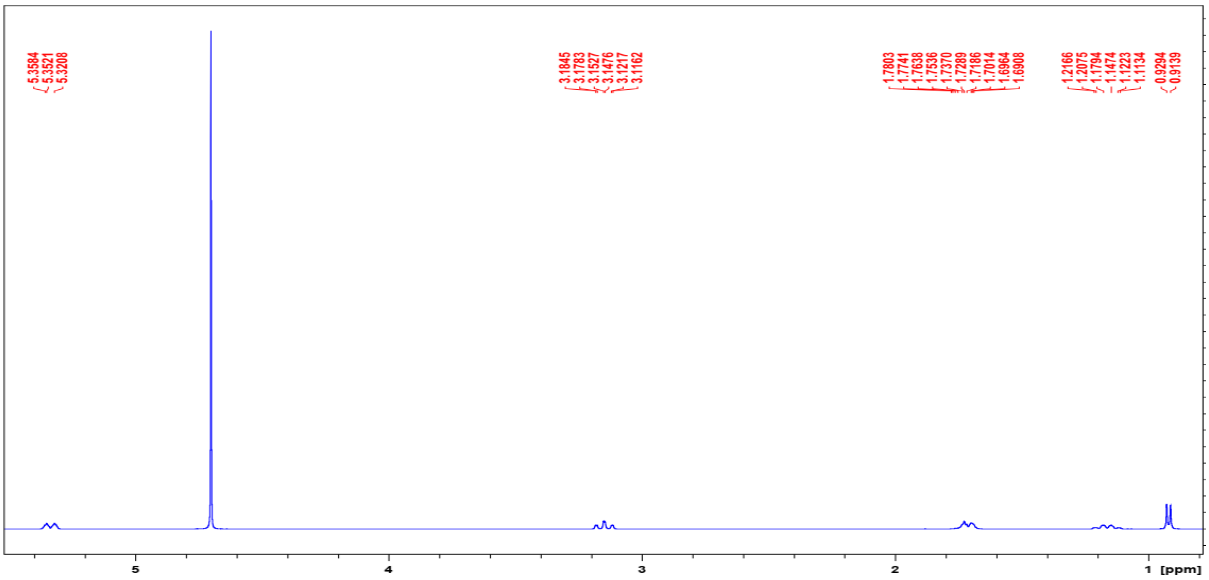

Figure S1. Proton NMR spectrum of 4-methylpiperidine dithiocarbamate

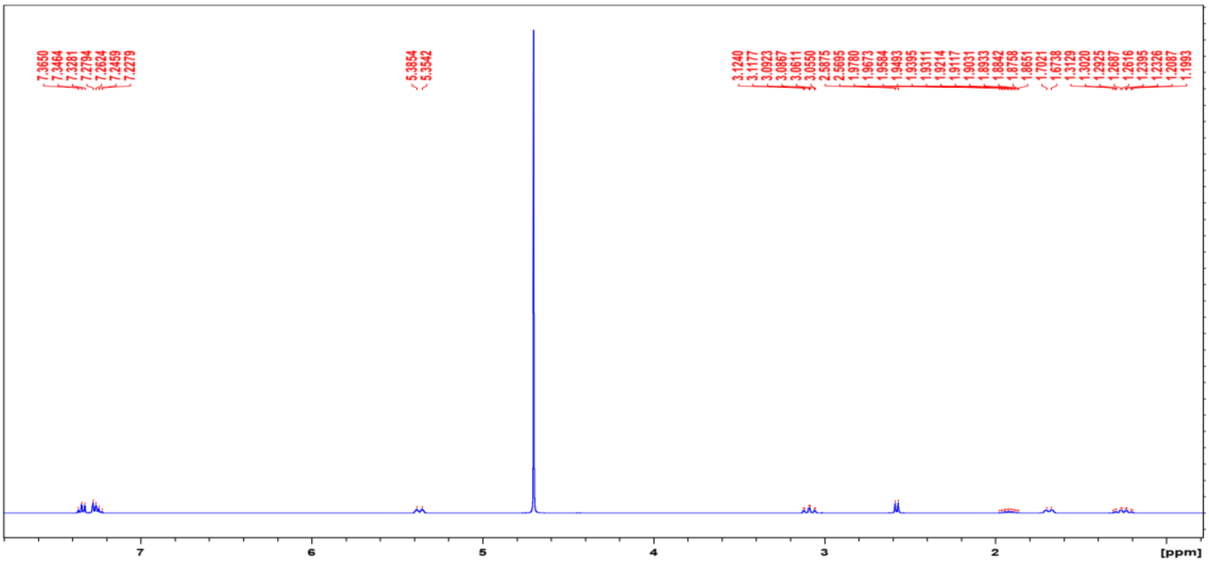

Figure S2. Proton NMR spectrum of 4-benzylpiperidine dithiocarbamate

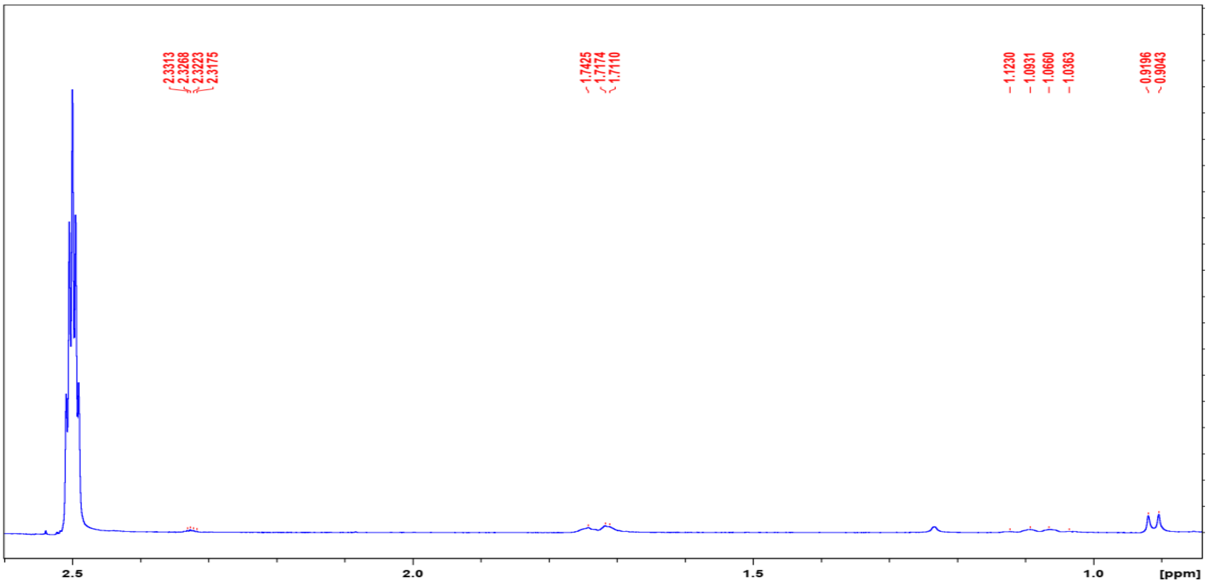

Figure S3. Proton NMR spectrum of bis(4-methylpiperidine-1-carbodithioato)-lead(II)

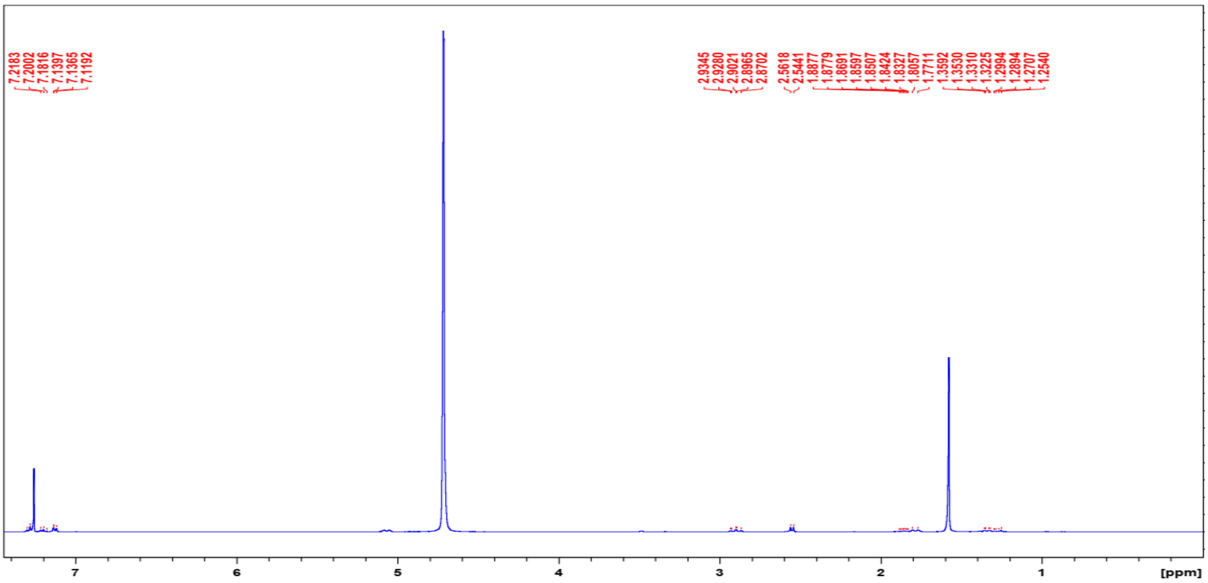

Figure S4. Proton NMR spectrum of bis(4-benzylpiperidine-1-carbodithioato)-lead(II)

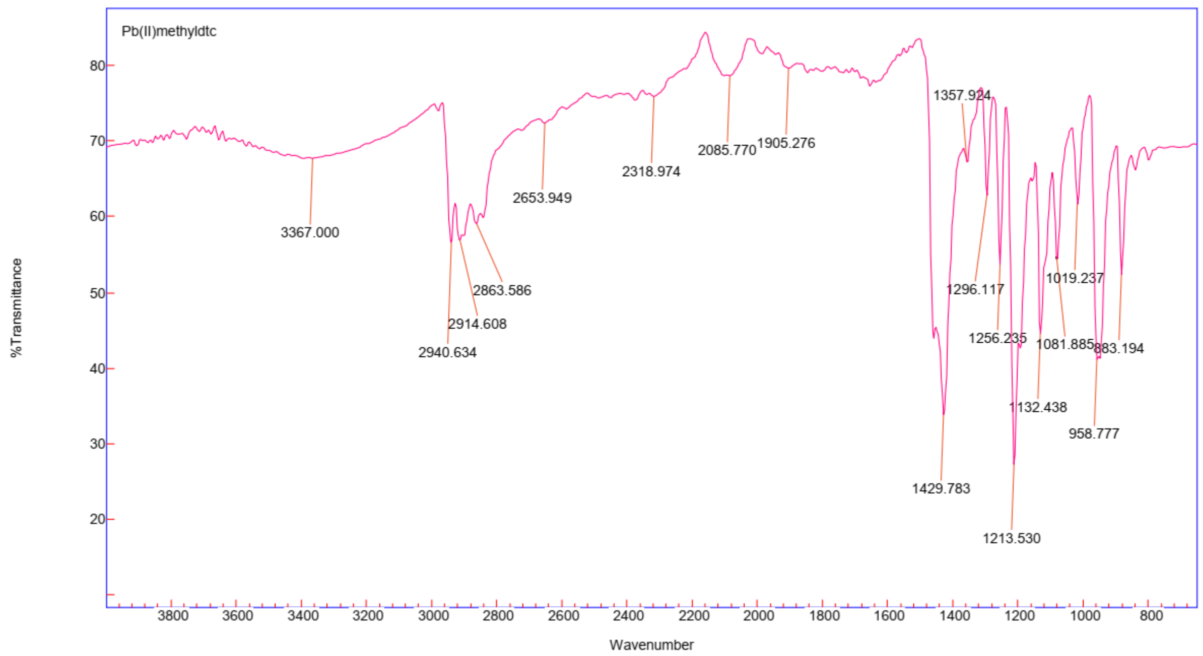

Figure S5. FTIR spectrum of bis(4-methylpiperidine-1-carbodithioato)-lead(II)

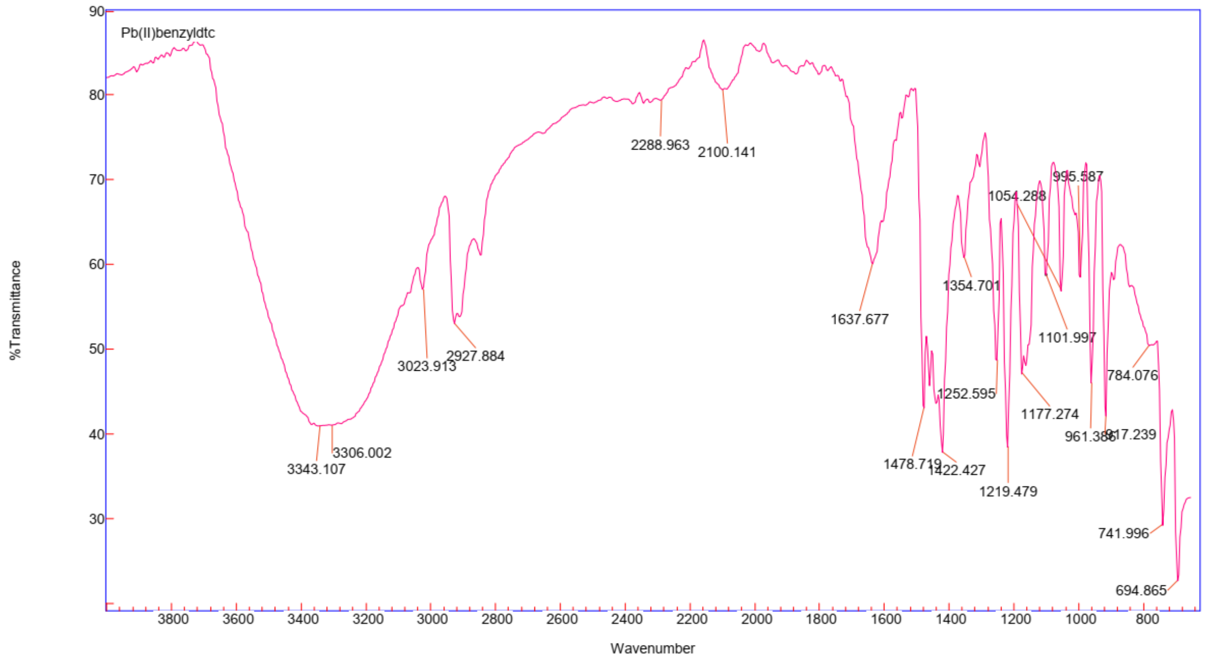

Figure S6. FTIR spectrum of bis(4-benzylpiperidine-1-carbodithioato)-lead(II)

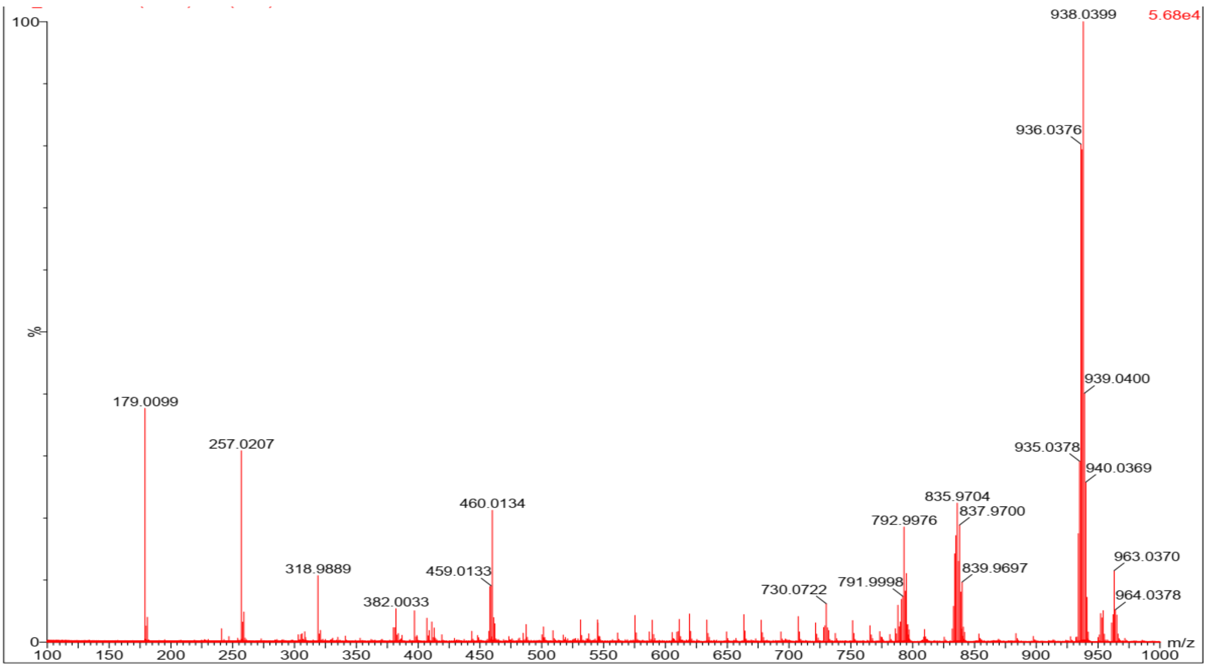

Figure S7. ToF mass spectrum of bis(4-methylpiperidine-1-carbodithioato)-lead(II)

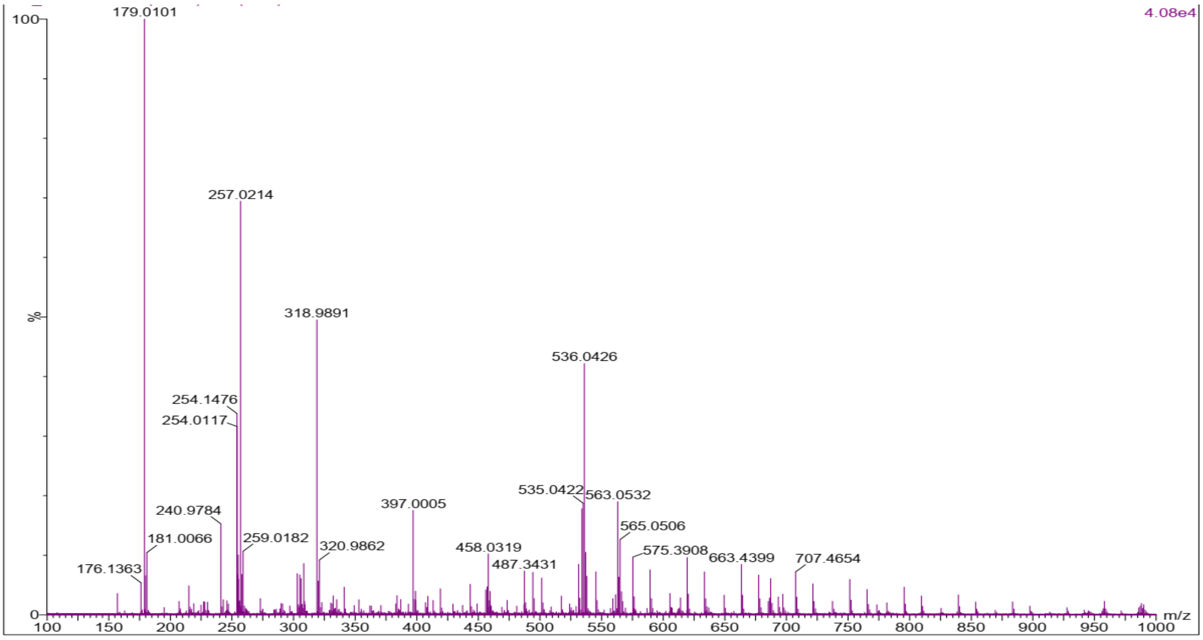

Figure S8. ToF mass spectrum of bis(4-benzylpiperidine-1-carbodithioato)-lead(II)

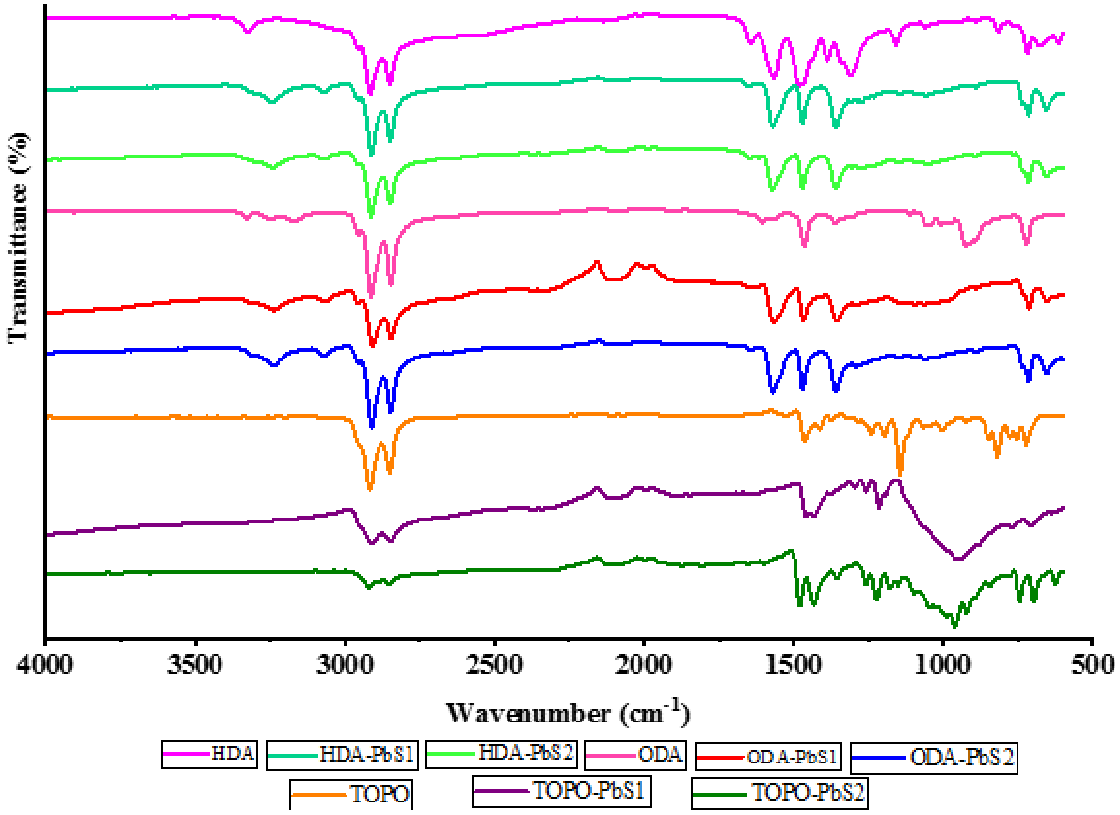

Figure S9. FTIR spectra of (HDA, ODA, and TOPO) capped as-prepared PbS nanoparticles

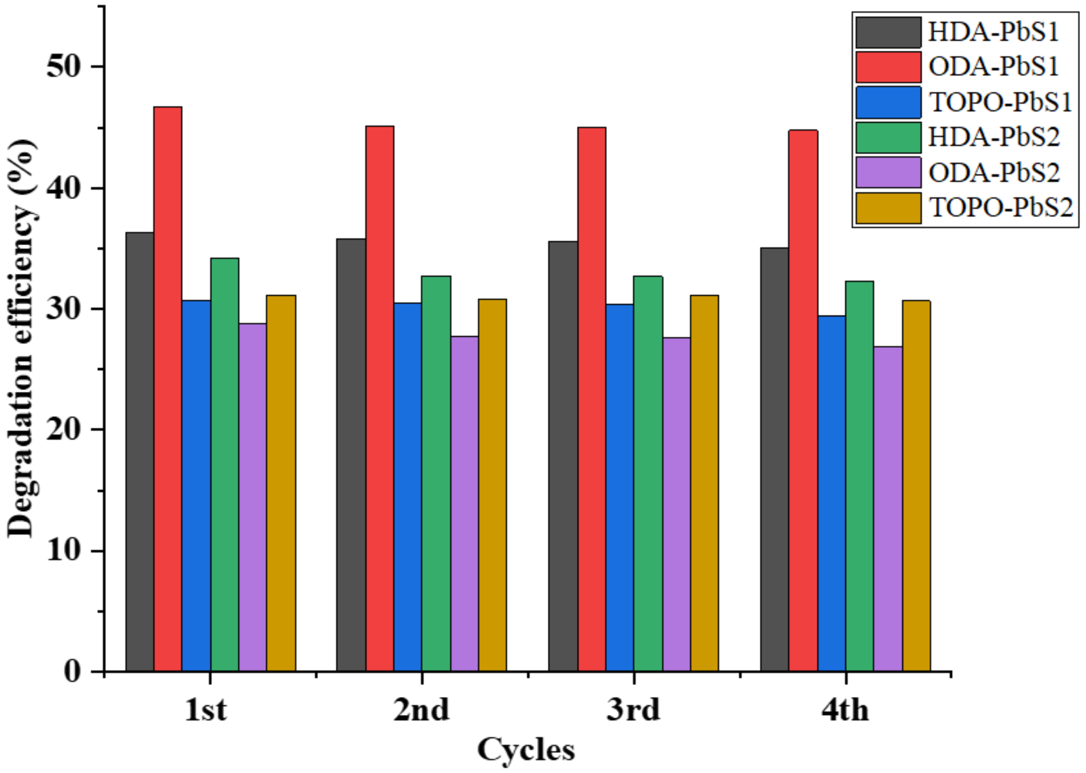

Figure S10. The reusability of PbS photocatalysts.

Table S1. Percentage (%) degradation for recyclability studies

	HDA-PbS1	ODA-PbS1	TOPO-PbS1	HDA-PbS2	ODA-PbS2	TOPO-PbS2
**1st**	36.28	46.71	30.71	34.22	28.70	31.17
**2nd**	35.84	45.08	30.49	32.76	27.75	30.83
**3rd**	35.57	45.06	30.34	32.71	27.64	31.09
**4th**	35.08	44.78	29.44	32.31	26.86	30.66
**% Change 1st & 4th**	1.20	1.93	1.27	1.91	1.84	0.51
